# Defect in recruiting effector memory CD8^+^ T-cells in malignant pleural effusions compared to normal pleural fluid

**DOI:** 10.1186/1471-2407-13-324

**Published:** 2013-07-02

**Authors:** Arnaud Scherpereel, Bogdan Dragos Grigoriu, Marc Noppen, Thomas Gey, Bachar Chahine, Simon Baldacci, Jacques Trauet, Marie-Christine Copin, Jean-Paul Dessaint, Henri Porte, Myriam Labalette

**Affiliations:** 1 INSERM Unit 1019, CIIL, Institut Pasteur de Lille, Lille, France; 2 Pulmonary and Thoracic Oncology Department, Hopital Calmette, CHRU de Lille, Lille cedex 59037, France; 3 Faculte de Medecine Henri Warembourg, University of Lille Nord de France, Lille, France; 4 Department of Pulmonary Diseases, University of Medicine and Pharmacy, Iasi, Romania; 5 Pulmonary Department, UZ Brussel, 101 Laarbeeklaan, Brussels, Belgium; 6 Department of Immunology, EA 2686, IFR114, CHRU de Lille, Lille, France; 7 Department of Pathology, CHRU de Lille, Lille, France; 8 Department of Thoracic Surgery, CHRU de Lille, Lille, France

**Keywords:** Pleura, Effusion, Tumor, Mesothelioma, Immunity, Lymphocytes

## Abstract

**Background:**

Malignant pleural effusions (MPE) are a common and fatal complication in cancers including lung or breast cancers, or malignant pleural mesothelioma (MPM). MPE animal models and immunotherapy trials in MPM patients previously suggested defects of the cellular immunity in MPE. However only few observational studies of the immune response were done in MPM patients, using questionable control groups (transudate…).

**Methods:**

We compared T cell populations evaluated by flow cytometry from blood and pleural effusion of untreated patients with MPM (n = 58), pleural metastasis of adenocarcinoma (n = 30) or with benign pleural lesions associated with asbestos exposure (n = 23). Blood and pleural fluid were also obtained from healthy subjects, providing normal values for T cell populations.

**Results:**

Blood CD4^+^ or CD8^+^ T cells percentages were similar in all groups of patients or healthy subjects. Whereas pleural fluid from healthy controls contained mainly CD8^+^ T cells, benign or malignant pleural effusions included mainly CD4^+^ T cells. Effector memory T cells were the main T cell subpopulation in pleural fluid from healthy subjects. In contrast, there was a striking and selective recruitment of central memory CD4+ T cells in MPE, but not of effector cells CD8^+^ T cells or NK cells in the pleural fluid as one would expect in order to obtain an efficient immune response.

**Conclusions:**

Comparing for the first time MPE to pleural fluid from healthy subjects, we found a local defect in recruiting effector CD8^+^ T cells, which may be involved in the escape of tumor cells from immune response. Further studies are needed to characterize which subtypes of effector CD8+ T cells are involved, opening prospects for cell therapy in MPE and MPM.

## Background

Malignant pleural effusions (MPE) are a common consequence of cancer with more than 150,000 cases of MPE per year are diagnosed in the USA [1]. MPE is found in up to 6% of patients with malignancy. Pleural metastases and/or MPE are mostly of lung cancer (15-30%), breast cancer (10-15%) or lymphomas origin. These patients have a poor prognosis, with a median survival of 3 to 6 months [1]. Among cancers associated with MPE, malignant pleural mesothelioma (MPM) was previously considered a rare tumor, but its incidence is increasing worldwide due to past (mostly occupational) exposure to asbestos. Outcome of patients with MPM remains poor despite recent treatment improvements [2]. Spontaneous immune response to mesothelioma seems to be important in counteracting tumor development. The immune system plays an important role in tumor biology, as it can delay mesothelioma progression [3], and immunotherapy has met with some success in previous and ongoing clinical trials [4-10]. In malignant effusions, the inflammatory processes and the immune responses induce the recruitment of cells into the pleural space [11]. The ability of T cell subsets at different differentiation stages to migrate to extra-lymphoid tissues as well as the immediate effector capacities of antigen-experienced T cell subsets, are heterogeneous [12]. Naïve and central memory (T_CM_) T cells constitutively express the CC-chemokine receptor (CCR7) permitting their traffic to lymphoid organs. Conversely, effector memory (T_EM_) and terminally-differentiated memory cells (T_TD_) have lost CCR7 expression, can traffic to peripheral sites, and are characterized by immediate effector capacities such as cytokine secretion and cytotoxic activity [13]. Depending on antigen load, either early or highly differentiated T-cells may exert the prominent protective capacity [14-17]. Previous studies never compared pleural cell populations from MPM patients with those of healthy subjects, but instead used various control groups such as pleural transudates, tuberculous effusions or ascites, none of which could provide accurate baseline values [18-20].

The aim of this study was to investigate the distribution of all four naïve and memory subsets composing the CD4^+^ and CD8^+^ T-cell pools in paired blood and pleural fluid from a large cohort of consecutive patients with MPM before any treatment. To better delineate specific changes in subset composition induced by mesothelioma, control groups included (a) otherwise healthy subjects with severe essential hyperhydrosis, (b) patients with benign lesions associated with asbestos exposure, and (c) patients with pleural metastases of carcinoma.

## Methods

### Patients

A total of 147 consecutive patients with a pleural effusion suspected of malignant pleural mesothelioma (MPM) were recruited in a prospective study involving pulmonary and thoracic surgery departments from 19 different hospitals in the North of France. Most of the patients had a documented history of exposure to asbestos; for detailed inclusion criteria, see reference [21]. Diagnosis was ascertained by trained pathologists on pleural biopsies obtained by thoracoscopy or thoracotomy, or occasionally by transthoracic CT guided biopsies. The final pathological diagnosis split the patients into three groups: 38 patients with pleural metastasis of adenocarcinoma (METS group), 30 patients with benign pleural lesions associated with asbestos exposure (BPLAE group), and 79 patients with malignant pleural mesothelioma (MPM group) (Table 1). No significant age difference was observed between these three groups. None of the recruited patients had received any anti-cancer treatment prior to inclusion. Some patients from the present cohort were also included in two previous publications on the diagnostic value of soluble mesothelin-related peptides and osteopontin in MPM [21,22].

**Table 1:** Demographic data, asbestos exposure of patients and diagnostic methods in recruited patients

	**METS (n = 38)**	**BPLAE (n = 30)**	**MPM (n = 79)**
**Age** years (mean ± SD)	65.8 ± 11.6	61.9 ± 11.3	64.3 ± 9.5
**Male gender** n (%)	21 (55.3%)	29 (96.7%)	64 (81%)
**Mesothelioma subtype** n (%)			
Epithelioid	-	-	60 (76%)
Mixed (biphasic) type			10 (12.6%)
Sarcomatoid			9 (11.4%)
**Primary tumor**			
Lung	14 (36.8%)	-	-
Breast	10 (26.3%)		
Digestive tract	3 (7.9%)		
Ovarian	2 (5.3%)		
Unknown	6 (15.8%)		
Other	3 (7.9%)		
**Diagnosed by:**			
Blind Pleural Biopsy	-	-	1 (1.3%)
Thoracoscopy	27 (71.1%)	21 (70%)	48 (60.8%)
Surgery	6 (15.8%)	9 (30%)	28 (35.4%)
Guided biopsy	5 (13.1%)	-	2 (2.5%)
**Asbestos exposure** n (%)			
Yes	11 (29%)	24 (80%)	61 (77.2%)
No	27 (71%)	5 (16.7%)	13 (16.5%)
Likely (but not confirmed)	-	1 (3.3%)	5 (6.3%)

*METS* pleural metastasis of adenocarcinoma, *BPLAE* benign pleural lesions associated with asbestos exposure, *MPM* malignant pleural mesothelioma.

In order to correctly interpret the lymphocyte immunophenotype profile of the different subgroups of patients we obtained normal pleural fluid by pleural lavage from subjects undergoing a stellate ganglion ablation under a thoracoscopic minimally invasive technique for severe essential hyperhidrosis. Fifteen otherwise healthy adults were recruited during the same period of time as the cancer patients presented above (using the same criteria as in reference [23]). These subjects had no history of prior pulmonary or pleural disease, and had a normal physical examination. Chest X-rays and complete pulmonary function testing confirmed that all were healthy except for the presence of essential hyperhydrosis at the palmar and/or axillar level. Paired simultaneous blood and pleural fluid samples were available for each subject in this group.

This study was performed under a research protocol approved by ethics committees from all involved institutions, and all patients gave an informed consent.

### Distribution of T cell subsets by multiparameter flow cytometry

Ethylene-diamine-tetraacetic acid anticoagulated samples of blood and pleural fluid were collected on the same day, sent to the laboratory within one hour, and analysed immediately upon arrival. Haemorrhagic pleural effusions were excluded from this study in order to avoid any bias generated by the contamination of pleural samples by blood.

Unprocessed whole blood and pleural samples were stained directly with fluorochrome-labelled monoclonal antibodies (concentrations according to the manufacturers’ instructions) followed by red cell lysis (UtiLyse reagent - Dako Chemicals) and two washings prior to acquisition. Staining was carried out by an automatic workstation (TQ-Prep) and analysis was performed on an Epics XL flow cytometer. All reagents and instrumentation were from Beckman Coulter (Fullerton, CA), with the exception of the monoclonal antibody to CCR7, from R&D systems (Minneapolis, MN). Relative numbers of CD4^+^ and CD8^+^ T lymphocytes were determined by a four-color assay using antibodies to CD3-PE, CD4-FITC, CD8-PC5, and CD45-PC7. Naive and memory subsets within CD4^+^ and CD8^+^ T-cell populations were determined by four colours staining with antibodies to CD4-PC7 and CD8-PC7, respectively, in combination with CD45RA-FITC, CCR7-PE, and CD28-PC5. Naive and memory T cell subsets were expressed as a percentage of the total number of CD4^+^ or CD8^+^ T cells respectively, by using the criteria published by Sallusto et al. [13]: naive (CD45RA^+^CCR7^+^), central memory (T_CM_, CD45RA^-^CCR7^+^), effector memory (T_EM_, CD45RA^-^CCR7^-^), and terminally-differentiated effector (T_TD_, CD45RA^+^CCR7^-^). Cells with potential regulatory function (T_reg_) were counted as CD4^low+^CD25^bright+^HLA-DR^Low^ gating after staining with CD4-PC7, CD25-PE and HLA-DR-FITC antibodies. Natural killer (NK) cells were analyzed using four colours staining with antibodies to CD3-PC7, CD8-PC5, CD16-PE and CD56-FITC.

### Statistical analysis

All data are reported as median and interquartile range (IQR) with the exception of age. Comparisons between groups were performed using a Kruskall-Wallis test and a non parametric ANOVA after rank transformation, as suggested by Conover [24]. A Bonferoni correction was applied for multiple comparisons in *post hoc* tests. Unavailable data (due to non availability of the biological probe or low quality or technical problems in flow cytometry analyses) were coded as missing. Statistical calculations were performed with SPSS statistical package (version 12.0 F, SPSS, Chicago, IL, USA).

## Results

### Distribution of lymphocyte subsets in paired blood and pleural fluid samples from healthy subjects

Reference values in pleural fluid were defined from pleural lavage fluids obtained during thoracoscopic treatment for severe essential hyperhydrosis of otherwise healthy adults (Table 2). NK cells (defined as CD3^neg^CD56^+^) were more abundant in pleural fluid than in peripheral blood (median of 16% versus 10%, respectively) but most pleural NK cells did not express the CD16 receptor, contrary to their peripheral blood counterparts, which were almost all CD16^+^ (median 6% versus 93%, respectively).

**Table 2:** Percentages of lymphocyte populations and their subset composition in pleural fluid assessed in healthy subjects and in patients. Results given as median (interquartile range)

	**METS**	**BPLAE**	**MPM**	**Healthy subjects**
**Lymphocyte populations**^**a**^				
Total CD3^+^ T-cells	80.5 (76–87.5)	85.2 (81–90.1)	77 (69–84)	85 (80–88)
CD4^+^ T-cells	63.2 (53.8-72)	63.5 (52.5-72)	54.2 (44–62.5)	30.0 (21–32)
CD8^+^ T-cells	19 (15–25)	22 (16.4-29)	25 (19.2-33.5)	53 (45–59)
CD4^+^/CD8^+^ ratio	3.8 (2.6-4.8)	3.2 (2.2-4.5)	2.2 (1.5-2.9)	0.59 (0.48-0.67)
Treg (CD4^low+^CD25^bright+^)^b^	4.4 (3–5.9)	4.4 (2.4-5.3)	3.1	2.2 (1.6-3.7)
NK cells (CD3^─^CD56^+^)	3.5 (2.2-7.5)	2.8 (2–4)	10 (3.4-12.5)	16.0 (12.4-24.1)
*→ % of CD16*^*+*^*NK cells*	*30 (20–59)*	*18 (1–35)*	*26 (14–54)*	*6% (3–8)*
**CD4**^**+**^**T cell subsets**^**b**^				
Naïve	22.7 (14.7-29)	25.7 (14.3-37)	11.2 (3.8-27)	1.6 (1–2.4)
T_CM_	47.4 (30–62)	53.5 (42.9-60)	40.5 (27.5-51)	13.1 (9.8-19.6)
T_EM_	21 (11.4-38.2)	18.6 (10.2-22)	19.2 (9.3-32)	85.1 (77.2-88.9)
T_TD_	0.6 (0.3-1.4)	1.3 (0.5-1.7)	0.8 (0.3-1.6)	0.7 (0.3-1)
**CD8**^+^**T cell subsets**^**b**^				
Naïve	15.2 (7.1-37.3)	26 (22–43.4)	11.2 (3.9-29.6)	1.4 (0.9-2.1)
T_CM_	22.1 (12–34.7)	27.4 (17.8-44)	11.6 (7.2-28.3)	2.3 (1.6-4.7)
T_EM_	19.7 (4.3-45.3)	16.1 (10.7-36)	18.6 (5.6-49.2)	84.7 (83.1-87)
T_TD_	12.7 (5.6-22.3)	13.7 (8–22.9)	7.4 (2.4-20.7)	9.5 (8.5-12.2)

^a^As a percentage of the total lymphocytes (defined as CD45^bright^, low side scatter).

^b^As a percentage of the total CD4^+^ or CD8^+^ T-cell population. The total may add up to slightly less or more than 100% because medians of individual subsets are given. the interquartile range for the MPM group could not be safely calculated due to the low number of cases; *T*_*CM*_ central-memory cells, *T*_*EM*_ effector memory cells, *T*_*TD*_ terminally-differentiated effector memory.

**Figure 1:**
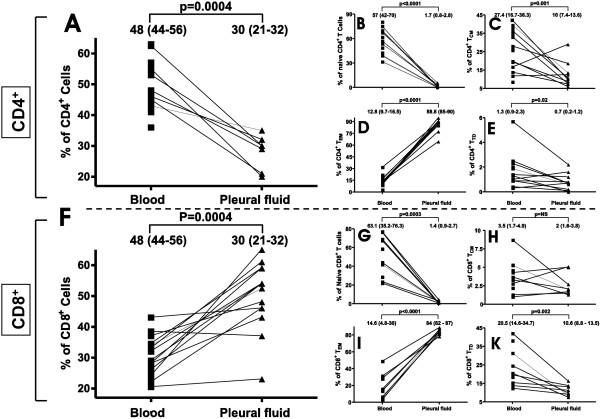
**Distribution of T cell populations in blood and pleural fluid of healthy subjects.** Distribution of CD4^+^ T cells (**A**), and CD8^+^ (**F**) T cells, as well as subtypes of naive (panels **B** and **G**), central memory - T_CM_ (panels **C** and **H**), effector memory - T_EM_ (panels **D** and **I**) and terminaly diferentiated - T_TD_ (panels **E** and **K**) in blood and pleural fluid. Numbers above each plot represent median values with interquartile range in squares.

T-lymphocytes were the most abundant cell population both in blood and in pleural fluid. As expected, CD4^+^ T cells represented the major T-cell population in peripheral blood, while CD8^+^ T cells constituted the major population in normal pleural fluid (Figure 1A and F), resulting in a CD4/CD8 ratio in pleural fluid (0.59 IQR 0,47-0,67) significantly lower than in blood (1.6 IQR 1.26- 2.18) (p < 0.001).

Compared to peripheral blood, the pleural fluid contained a very low proportion of naïve CD4^+^ and CD8^+^ T cells (Figure 1B and G). The main subsets in the pleural fluid had an effector-memory phenotype (>80%) within both CD4^+^ and CD8^+^ T-cells subsets (Figure 1D and I). The proportion of terminally-differentiated CD8^+^ T cells was lower in pleural fluid than in blood, and terminally differentiated CD4^+^ T cells were rare (less than 6%) in both sample sources. Regulatory T-cells, defined as the CD4^low+^CD25^bright+^ HLA-DR^Low^ population [25], were scant and their percentages were not significantly different between blood and normal pleural fluid.

### Distribution of lymphocyte subsets in pleural fluid from patients with pleural effusions

#### Natural killer (NK) cells

**Figure 2:**
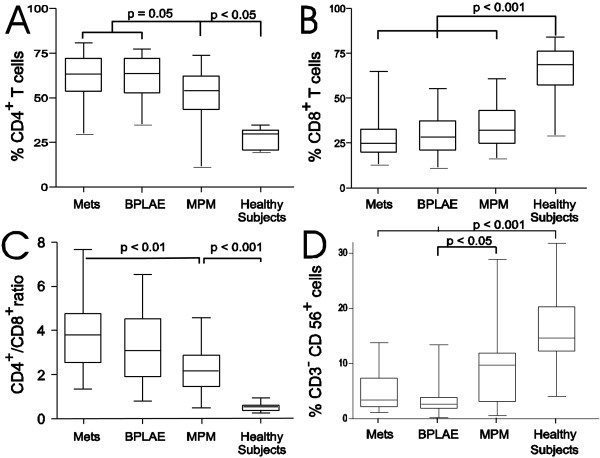
**Distribution of populations in pleural fluid of patients vs. healthy subjects.** Distribution of CD4^+^ (**A**) or CD8^+^ (**B**) T cell subsets, as well as the CD4^+^/CD8^+^ T (**C**) cell ratio and NK cells )**D**) in the pleural fluid of different groups of patients (pleural metastasis of adenocarcinoma = METS group), patients with benign pleural lesions associated with asbestos exposure = BPLAE group), and patients with malignant pleural mesothelioma = MPM group) vs. healthy subjects. Horizontal line in boxes represent median values, box limits represent 25th (Q1) and 75th (Q3) centiles and whiskers minimal and maximal values.

In pleural fluid, the relative proportion of NK cells was much lower in all groups of patients than in healthy controls (p < 0.001), although patients with mesothelioma (MPM group) kept relatively more pleural NK cells than in the BPLAE and METS groups (Figure 2D and Table 2). This low proportion of NK cells within pleural fluid of patients contrasted with the elevated proportion of NK cells in the peripheral blood of the same patients with median values (IQR) of 18.6% (11.6-33.5), 13.5% (7.8-22.3), 15.1% (9.3-22.5) for METS, BPLAE and MPM groups respectively compared to 9,6% (7.05-14.6) for the healthy control group. In all patients, pleural NK cells expressed more often the CD16 molecule: medians of 30%, 18%, and 26% of total NK cells in METS, BPLAE and MPM patients respectively, versus 6% in healthy controls (Table 2).

#### Total CD4^+^ and CD8^+^ T cell counts

The contribution of T-cells to the total lymphocyte count was significantly higher in pleural fluid than in blood for all groups of patients (p = 0.007 or less). Healthy subjects also had mainly CD8^+^ T cells in their pleural fluids, while patients with benign or malignant pleural effusion had mainly CD4^+^ T cells: the median pleural CD4^+^/CD8^+^ ratio in healthy subjects was 0.59 while in patients it always exceeded 2.2 (Figure 2A-C). However, the relative proportions of CD4^+^ and CD8^+^ T cells in paired blood were very similar in healthy subjects and in METS, BPLAE, or MPM patients (data not shown). Furthermore, CD4^+^ T-cells in the pleural fluid of patients with either metastatic carcinomas or benign pleural lesions associated with asbestos exposure tended to be relatively more abundant than in MPM patients (Figure 2A).

Data for cells with a CD4^+low^CD25^bright+^ phenotype were available only for 23 paired specimens (12 with METS, 6 with BPLAE, 5 with MPM). Percentages of this CD4^low+^CD25^bright+^ population were low in the blood and the pleural fluid in each group, accounting for less than 5% in virtually all specimens, without significant difference among the 4 groups studied (p = 0.17).

#### Naïve and memory T-cell subsets

**Figure 3:**
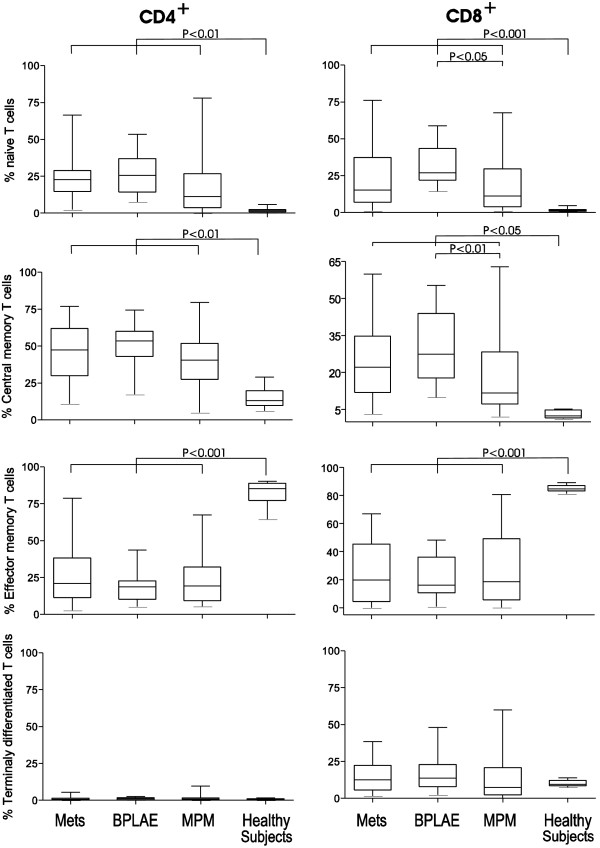
**Distribution of T cell subpopulations in pleural fluid of patients vs. healthy subjects.** Distribution of naive, central memory - T_CM_, effector memory - T_EM_, and terminally differentiated - T_TD_ T cells among CD4^+^ and CD8^+^ T cell populations. Results are expressed as percentage of the total number of CD4^+^ and CD8^+^ T cells respectively.

The distribution of naïve and memory CD4^+^ or CD8^+^ T-cell subsets in pleural fluid differed markedly between patients and healthy controls. Surprisingly, CCR7^+^ naïve and central memory T-cells represented the main subsets within the abundant CD4^+^ T-lymphocyte population in the pleural fluid of all patients (Figure 3, Table 2). Within the less abundant CD8^+^ T-cells, effector memory CD8^+^ T cells were significantly less represented than in normal pleural fluid and percentages in patients’ pleural fluid were lower in paired blood from all patients’ groups (p < 0.001 or less for all comparisons). Pleural fluid from patients with MPM contained fewer naïve and central memory CD8^+^ T-cells than in asbestos-exposed patients without mesothelioma (p < 0.05 or less).

## Discussion

It is widely admitted that T cells and NK cells are key players in anti-tumour immunity, although progressing tumours can ultimately escape immune control. The assessment of T cell subpopulations and NK cells in blood and pleural fluid could be useful for the understanding of the mechanisms involved in leukocyte recruitment in malignant pleural effusions and may help understanding their involvement in anti-tumor immunity. We report here the relative distribution of these populations in patients with MPM and compared it with values obtained from patients with pleural metastases of various carcinomas as done previously [23,26], but also from patients with benign pleural involvement related to asbestos exposure and for the first time healthy subjects. This permitted us to more precisely analyze circulating and tumor environment-associated immune cells in patients with malignant pleural effusions. To our best knowledge few studies have specifically reported CD4^+^ et CD8^+^ T cell subpopulations in malignant pleural effusions [19,27,28].

Our results show for the first time that the pleural fluid from healthy subjects exhibits a significant different CD4^+^/CD8^+^ T cell ratio not only compared to normal blood value but also with pleural fluid from all groups of patients, as suggested by other authors [20,27,29]. This distribution of the CD4^+^/CD8^+^ T cells in healthy subjects appeared also very similar to the one observed in peritoneal fluid from healthy women [30]. By contrast, we found that in all our patients there was a defect in recruiting CD8^+^ in the abnormal pleura, as previously reported by several authors [27,28,31,32]. Interestingly this pleural recruiting defect was slightly less important (but statistically significant) in MPM cases than in patients with pleural metastases (METS) or with benign pleural lesions associated to asbestos exposure (BPLAE).

The first bias to eliminate in order to correctly understand these results is a potential contamination of the pleural samples with blood. This was unlikely because we excluded all hemorrhagic pleural effusions. Furthermore, the distribution of blood T cell subsets in our patients was similar to the one found in healthy subjects of the same age [26], except the lower percentages of naive CD8^+^ T cells in the METS and the MPM groups.

Immune response plays an important role in pleural tumours, and the development of a CD8^+^ mediated immune response have been associated with treatment success in animal models as well as in humans [33-36]. Here we show that this less abundant CD8^+^ T cells compartment is not of the effector-memory phenotype, as in healthy subjects. Prado-Gracia et al. previously reported that only a low percentage of this T CD8^+^ cell population expressed perforin, an important mediator of cell-mediated cytotoxicity [28]. In our study, the percentages of total CD8^+^ T cells or of CD8^+^ T cell subsets in blood or pleural effusion assessed before any treatment were not associated with survival in a Cox proportional hazard model (data not shown). However, it would be very interesting to study the kinetics of CD8^+^ T cell populations in patients with malignant pleural effusions of all types under different therapies, and its potential relation with patient outcome as previously reported in animal tumor models [29,30].

In all groups of patients, the enrichment of pleural fluid with CD4^+^ T cells was due to an increase in the percentages of central memory and naive CD4^+^ T cell subsets. This was an unexpected finding since these cells should theoretically rather be found circulating from blood to the lymphoid organs. The same T cells subpopulations were predominant among the CD8^+^ T cells in the pleural fluid while one would normally expect an increase in effector memory and terminally differentiated CD8^+^ T cells as we found in healthy subjects. Compared to benign pleurisies associated with asbestos exposure, MPE were associated with a decrease in CD8^+^ naïve and central memory T cells. But this was not associated with an increase of effector memory and terminally differentiated CD8^+^ populations. These results are in accordance with the results published by Prado-Garcia et al. and by Atanackovic et al. [27,28] who reported an increase of non effector CD4^+^ and CD8^+^ subsets as well as with the data of Aguiar et al. [19] who found a diminishing Naïve T cell population along with an increase of memory T cells in pleura of patients with MPE.

Possible explanations for these findings could relate to different mechanisms. First a defect in local recruitment of effector T cells in target organs or MPE may be due to changes of chemo-attractant signals by the tumor [37,38]. A second hypothesis could be that effector cells rapidly undergo apoptosis which generate a higher T cell turnover with accumulation of central memory cells and a depletion in effector cells compartment [19,39]. A third hypothesis would be a deficit in the expression of the ζ chain [40-43] which is important in the signal transduction via the T cell receptor. This could results in a defect in activating T cells and thus a generation of a lower number of effectors.

We also found in normal pleural fluid a lower proportion of natural regulatory T cells (T_reg_) than in the pathological pleural effusions. These results are consistent with previous reports [44-47]. However this accumulation of T_reg_ seems to be extremely moderate and related more to the pleura itself than to the type of pleural disease. However, firm conclusions are difficult to extract due to the very limited number of patients in each subgroup. It has also to be taken into account that human malignant mesothelioma tissue contains not only regulatory T cells but also cytokines and chemokines suppressing an efficient anti-tumor immune response and promoting angiogenesis [38,47-49].

Natural killer cells are also a very important component of the anti-tumor immune response. In accordance with previous reports [27,50], we found that despite a higher percentage of circulating NK cells in patients with pleural malignancies (or even in BPLAE patients) than in healthy subjects, there was a defect in recruiting NK cells in the malignant pleural effusions. This defect seemed to be less important in MPM patients. In the literature, NK cells which express the low affinity Fc receptor, FcγRIII (with a CD56^+(low)^CD16^+^ phenotype) have mostly a cytotoxic function [51] while the CD56^+(high)^CD16^-^ NK cells mostly produce various cytokines [52]. In our patients, the defect of pleural NK cells mainly concerned mainly the CD16^neg^ cells.

There are two crucial and still unsolved questions. Are these findings in MPE correlated with local T cell populations in tumor tissue? Can these changes be therapeutically manipulated in order to obtain a better response to treatment by transforming local immunologic tolerance into anti-tumor immune response? Recently CD8+ tumor infiltrating lymphocytes (TIL) have been associated with a better survival in resected MPM patients [53].

## Conclusion

The goal of the present study was to describe tumor associated lymphocytes in MPE and compare them for the first time with pleural values from healthy subjects. We confirmed that there is a defect in recruiting CD8^+^ effector T cells in MPE. Several new treatments, including gene and cell therapies which target anti-tumor immune response, are now tested in patients with MPM or pleural metastases and should investigate this matter. These results suggest that current or future treatments for MPM or pleural metastases might be more effective if combined with immunotherapy to circumvent the local immunosuppression [54,55].

## Competing interests

The authors declare that they have no competing interests.

## Authors’ contributions

AS conceived the design and coordinated the study, and wrote the manuscript. BDG participated in the design of the study, performed the statistical analysis, and helped to draft the manuscript. MN participated in the patients recruitment and the samples collection. TG, BC, and SB collected clinical data from patients, and helped to draft the manuscript. VM. JT carried out the immunoassays. MCC and JPD revised the manuscript critically for important intellectual content. HP participated in the patients’ recruitment, and helped to draft the manuscript. ML coordinated the biological part of the study, and helped to draft the manuscript. All authors read and approved the final manuscript.

## Authors’ information

AS is the national coordinator of “MESOCLIN”, the French clinical expert network for the management of malignant pleural mesothelioma, and the elected secretary of the Thoracic Oncology Assembly (TOA) of the European Respiratory Society (ERS). Bogdan Dragos Grigoriu is the elected chair of the “pleural and mediastinal malignancies” group of the TOA of the ERS. Marc Noppen is an international well-known expert on pleural diseases. Marie-Christine Copin is the head of the Pathology Department in Lille, and a member of the international expert of the French pathology expert network “MESOPATH”. Myriam Labalette is the head of the Immunology Lab of the University Hospital (CHRU) of Lille.

## Pre-publication history

The pre-publication history for this paper can be accessed here:

http://www.biomedcentral.com/1471-2407/13/324/prepub
